# Fludrocortisone for Refractory Hypotension in a Hospitalized Dialysis Patient: A Case Report

**DOI:** 10.7759/cureus.92768

**Published:** 2025-09-20

**Authors:** Fnu Veerban, Meenakshi Kurup, Ibrahim Mohamed, Moro Salifu, Samy I McFarlane, Muhammad Azhar, Isha Puri, Mary Mallappallil

**Affiliations:** 1 Department of Internal Medicine, Division of Nephrology, State University of New York Downstate Health Sciences University, Brooklyn, USA; 2 Department of Internal Medicine, Division of Nephrology, NYC Health + Hospitals Kings County Hospital Center, Brooklyn, USA

**Keywords:** acute kidney injury, continuous renal replacement therapy (crrt), drug-induced acute interstitial nephritis, fludrocortisone, heart failure with reduced ejection fraction, hemodialysis, intradialytic hypotension, volume overload

## Abstract

We present the case of a 74-year-old woman with stage 4 chronic kidney disease (CKD) who developed acute kidney injury (AKI) due to drug-induced acute interstitial nephritis (AIN) following exposure to piperacillin-tazobactam 2.25 grams three times a day and ursodiol 600 mg daily. Despite corticosteroid therapy, her renal function declined, necessitating the initiation of maintenance hemodialysis. During a subsequent admission, she developed persistent intradialytic hypotension (IDH), with her systolic blood pressure ranging from 60 to 70 mm Hg and diastolic blood pressure ranging from 35 to 45 mm Hg. Laboratory evaluation revealed a low morning cortisol level, consistent with adrenal insufficiency secondary to prior steroid use. ACTH stimulation testing was not performed due to the patient’s clinical instability; however, recent corticosteroid use, persistent hypotension, low cortisol levels, and volume overload provided sufficient evidence of secondary adrenal insufficiency to guide management. The patient was commenced on stress-dose hydrocortisone and midodrine, but hypotension persisted. The addition of fludrocortisone resulted in sustained hemodynamic improvement by enhancing sodium and water retention, as well as improving vascular tone. Her blood pressure improved, with systolic values ranging from 98 to 105 mm Hg and diastolic values from 55 to 65 mm Hg. This enabled successful hemodialysis tolerance over the following 2.5 months. Informed consent for publication of this case and its accompanying details was obtained from the patient’s family. Fludrocortisone, commonly used in ambulatory dialysis, proved valuable in this case by demonstrating efficacy in the acute inpatient setting, initiated at a dose of 0.1 mg and titrated to 0.2 mg daily. It underscores the therapeutic role of fludrocortisone in managing refractory hypotension due to adrenal insufficiency, even in patients with minimal residual renal function, and emphasizes the importance of early recognition and treatment of corticosteroid-induced adrenal suppression in CKD patients.

## Introduction

Adrenal insufficiency is a potentially life-threatening complication of corticosteroid therapy, particularly in patients with chronic kidney disease (CKD) or those receiving dialysis [1]. Persistent hypotension during hemodialysis (intradialytic hypotension, IDH) presents a common and challenging complication, occurring in up to 20-30% of dialysis sessions, and often limits the efficacy and safety of treatment. Fludrocortisone, a synthetic mineralocorticoid, has been shown to improve refractory hypotension in the inpatient and outpatient setting by enhancing sodium retention and increasing vascular tone. Its use in the ambulatory dialysis setting has demonstrated efficacy in raising blood pressure, but its inpatient use remains rare and underreported [2].

We report the case of a patient with stage 4 CKD who developed acute kidney injury (AKI) due to drug-induced acute interstitial nephritis (AIN), followed by adrenal insufficiency secondary to corticosteroid therapy. The addition of fludrocortisone was instrumental in achieving hemodynamic stability and facilitating successful maintenance hemodialysis. This report highlights the clinical relevance of early recognition of adrenal suppression, the therapeutic potential of fludrocortisone in the inpatient setting, and the importance of careful monitoring for associated electrolyte and volume-related complications.

## Case presentation

A 74-year-old woman with a past medical history of type 2 diabetes mellitus, hypertension, hyperlipidemia, peripheral artery disease, iron deficiency anemia, heart failure with reduced ejection fraction (~40%), primary biliary cirrhosis, and stage 4 CKD (baseline creatinine ~2.1 mg/dL; eGFR ~21 mL/min/1.73 m²) presented with progressive bilateral lower extremity edema, non-productive cough, and exertional dyspnea. She denied orthopnea, fever, or chest pain. On presentation, her vital signs included a blood pressure of 105/61 mm Hg, heart rate of 81 bpm, respiratory rate of 19/min, and temperature of 98°F. Home medications included insulin glargine, insulin lispro, sitagliptin 25 mg, atorvastatin 40 mg, aspirin 81 mg, apixaban 2.5 mg, calcium acetate 667 mg, and ursodiol. See Table 1 for the initial laboratory values on presentation.

**Table 1:** Initial laboratory values at presentation Urinalysis pH = 6, specific gravity = 1.020, WBC = 50-100, RBC = 2, protein = 100 Cr: creatinine; BUN: blood urea nitrogen; eGFR: estimated glomerular filtration rate; WBC: white blood cell count; RBC: red blood cell; AST: aspartate aminotransferase; ALT: alanine aminotransferase; ALP: alkaline phosphatase; CO₂: carbon dioxide

Laboratory test	Patient value	Reference range
Sodium	141 mEq/L	135-145 mEq/l
Potassium	4.3 mEq/L	4.2-5.5 mEq/L
Chloride	95 mEq/L	98-106 mEq/L
Co2	28 mEq/L	22-24 mEq/L
Creatinine (Cr)	2.9 mg/dl	0.6-1.1 mg/dl
Baseline Cr	2.1 mg/dl	0.6-1.1 mg/dl
BUN	74 mg/dl	24 mg/dl
eGFR	16 ml/min/1.73	90 ml/min/1.73
Hemoglobin	8.6 g/dl	13-15 g/dl
WBC	5.6 x10^9/L	4-11 x 10^9^/L
Platelets	168 x10^9/L	150-300 x 10^9^/L
Eosinophils	11 %	<5%
Calcium	8.5 mg/dl	8.5-10 mg/dl
Phosphorus	5.0 mg/dl	2.5-4.5 mg/dl
Albumin	3.3 g/dl	4.2 g/dl
Urine protein	279/214 1.3 g/day	0-14 mg/dl
Bilirubin	0.5 mg/dl	0.1-1.2 mg/dl
AST	25 U/L	10-40 U/L
ALT	15 U/L	10-45 U/L
ALP	222 U/L	30-120 U/L

The patient experienced an acute kidney injury on CKD, with her serum creatinine rising from 2.1 to 2.9 mg/dL and eGFR falling to <15 mL/min/1.73 m². Urinalysis showed proteinuria, pyuria, and an increased urine protein-to-creatinine ratio (0.7 to 1.3 g/day). Peripheral eosinophilia was noted. Renal ultrasound revealed bilaterally echogenic kidneys without hydronephrosis, consistent with chronic parenchymal disease. A diagnosis of drug-induced AIN was made, and the patient was commenced on 60 mg prednisone daily. Despite corticosteroid therapy, her renal function worsened further from a serum creatinine of 2.9 rising to 6.9 mg/dL and BUN 215 mg/dL. Her clinical assessment indicated worsening volume overload, with the development of 2+ pitting edema and escalating oxygen requirements to maintain her oxygen saturation levels. She responded poorly to diuresis with intravenous furosemide 100 mg twice daily and bumetanide 2 mg twice daily. Hemodialysis was initiated, and she was discharged to an outpatient dialysis center on a prednisone taper over two weeks.

The patient was readmitted within days of discharge with persistent hypotension and volume overload, resulting in poor tolerance of hemodialysis. Her systolic blood pressure ranged from 70 to 85 mm Hg and diastolic pressure from 38 to 53 mm Hg. Endocrine evaluation demonstrated low cortisol levels, indicating adrenal insufficiency attributable to recent short-term corticosteroid exposure, underscoring that even brief courses can suppress the hypothalamic-pituitary-adrenal axis (see Table 2).

**Table 2:** Endocrine and electrolyte parameters at readmission

Laboratory test	Patient value	Reference range
Morning cortisol	5.23 microgram/dl	6.20-19.40 microgram/dl
Potassium	4.9 mEq/L	3.5-5.0 mEq/L
Magnesium	2.1 mEq/L	1.8-2.2 mEq/L

The patient was started on intravenous hydrocortisone 50 mg every six hours and midodrine for persistent low diastolic pressures. Due to ongoing volume overload and low systolic blood pressure, continuous renal replacement therapy (CRRT) was initiated to optimize her fluid status. Hemofiltration, a convective therapy used in her case, has been reported to reduce IDH and improve hemodynamic stability [3].

As her condition stabilized, she was transitioned to intermittent hemodialysis and started on oral hydrocortisone 25 mg and midodrine. Despite supportive measures, she remained unable to tolerate hemodialysis owing to refractory IDH, with her systolic blood pressure falling to 68-85 mm Hg and diastolic pressure to 34-57 mm Hg. Multiple strategies, including adjusting dialysate temperature, increasing dialysis frequency, modifying sodium levels, and withholding hypotensive medications, were attempted without success [4].

**Figure 1:**
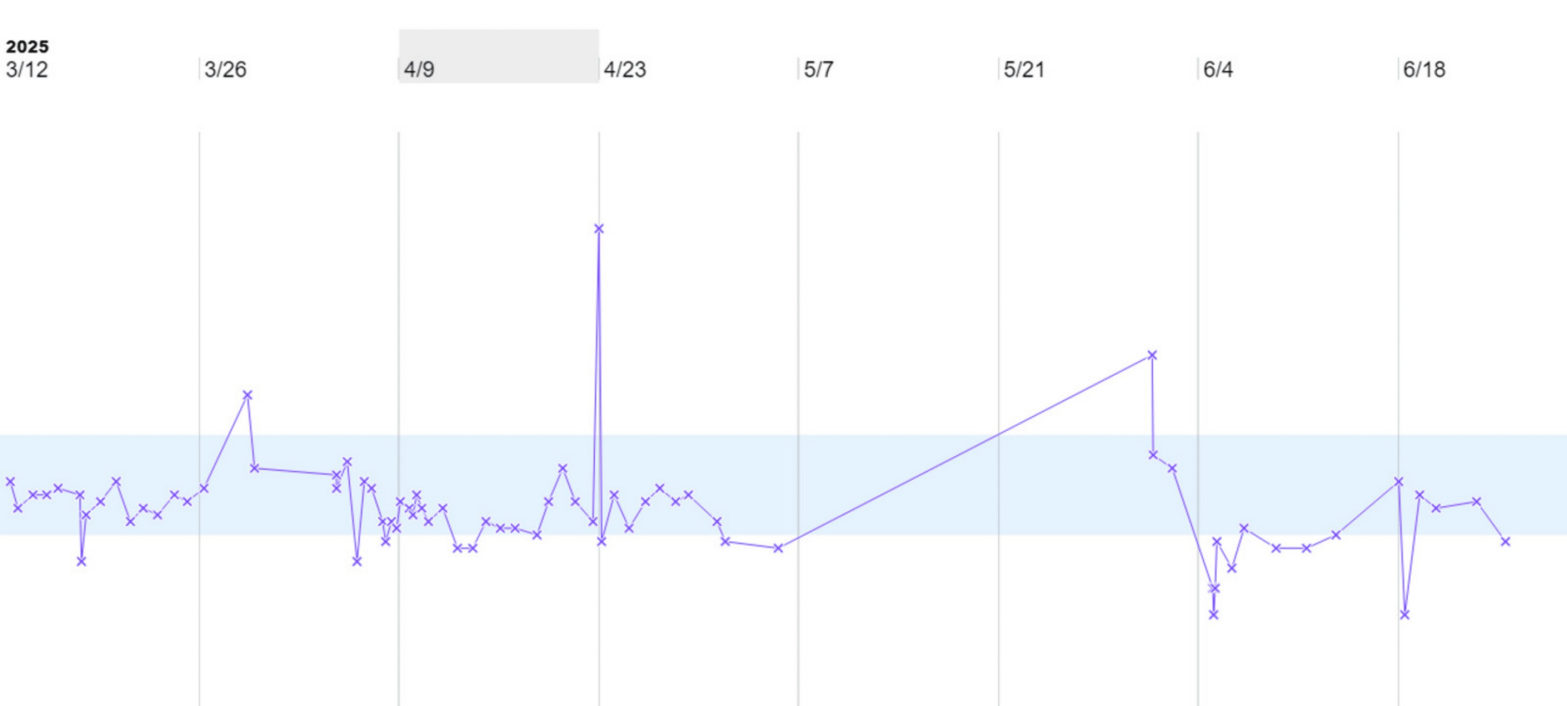
Trend of serum potassium levels during hospitalization The area highlighted in blue corresponds to the reference range for potassium and magnesium.

**Figure 2:**
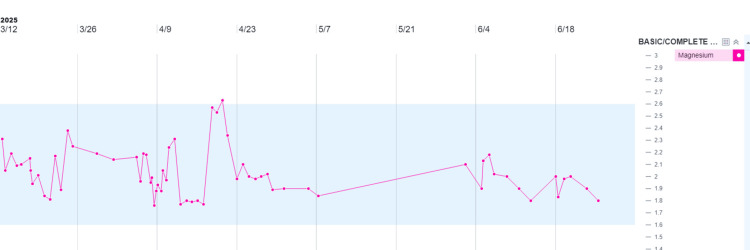
Trend of serum magnesium levels during hospitalization The area highlighted in blue corresponds to the reference range for potassium and magnesium.

Initiation of fludrocortisone 0.2 mg daily resulted in significant improvement in blood pressure control and dialysis tolerance. Despite the initiation of hemodialysis, the patient retained some residual renal function, evidenced by persistent hypokalemia with normal magnesium levels. Electrolytes were closely monitored (see Figures 1, 2). 

Over the subsequent two and a half months, the patient tolerated hemodialysis without recurrent IDH or other complications. She was later readmitted with sepsis secondary to pressure ulcers. Following a multidisciplinary discussion, her code status was changed to do not resuscitate/do not intubate (DNR/DNI), and she expired during her hospitalization.

## Discussion

Under routine circumstances, blood pressure typically declines after hemodialysis, largely influenced by the volume of fluid removed during treatment [5]. In this case, however, the patient experienced a significant drop in blood pressure during dialysis, which limited ultrafiltration and exacerbated volume overload. This atypical pattern raised concern for unrecognized adrenal insufficiency and possible autonomic dysfunction, both of which are common in patients with long-standing diabetes [2].

Despite discontinuation of hypotensive medications (including hydralazine 50 mg three times a day and metoprolol succinate extended release 25 mg daily), optimization of ultrafiltration rates, increasing dialysis frequency, dialysate temperature control, and the use of hydrocortisone and midodrine, the patient continued to experience symptomatic IDH. A notable aspect of this case was the successful use of fludrocortisone in the acute inpatient setting to support blood pressure and enable effective dialysis.

Fludrocortisone, a synthetic mineralocorticoid with aldosterone-like properties, was titrated to 0.2 mg daily. It promoted intradialytic blood pressure stability and intravascular volume expansion by enhancing sodium and water retention and improving vascular responsiveness. It also maintained the patient's blood pressure without worsening her volume status [6]. The patient’s residual renal function, evident by an eGFR of 15 mL/min/1.73 m² at dialysis initiation and short dialysis vintage, likely contributed to the effectiveness of this therapy. With fludrocortisone, she tolerated maintenance hemodialysis for two and a half months without requiring vasopressors or frequent prescription adjustments.

While fludrocortisone is not commonly used in dialysis populations, its role may be underrecognized in patients with adrenal suppression or suspected autonomic dysfunction [7]. Those most likely to benefit are individuals with refractory IDH who have failed optimized non-pharmacologic and first-line pharmacologic measures and demonstrate features of neurogenic or diabetic autonomic hypotension [8,9]. Fludrocortisone has been trialed in chronic, stable outpatient hemodialysis settings to prevent IDH [10]. Its use has also been reported in ambulatory dialysis patients to support blood pressure and manage hyperkalemia, with variable efficacy [2,11-13]. In addition, it is used in managing neurogenic hypotension in Parkinson’s disease and diabetic postural hypotension [8,9]. Fludrocortisone’s use in treating hyperkalemia in advanced CKD not yet on dialysis has been described, with mixed outcomes [6].

However, caution is required. Reported side effects include cardiovascular complications such as arrhythmia, chest pain, and worsening heart failure, particularly concerning patients with existing cardiac disease. Electrolyte disturbances, particularly hypokalemia, must be closely monitored due to associated risks such as muscle weakness, fatigue, and cardiac arrhythmia. Due to the potassium-lowering effect of the medication, it has been used in advanced CKD patients not yet on dialysis to treat hyperkalemia [14]. Psychiatric effects such as anxiety, mood swings, depression, and even psychosis have also been reported, as well as increased susceptibility to infections [15].

Fludrocortisone likely contributed to hemodynamic stability through several mechanisms: by promoting renal sodium and water reabsorption, enhancing vascular sympathetic tone, and counteracting nitric oxide-mediated vasodilation, thereby improving vascular resistance and mitigating refractory IDH even in the setting of CKD and baseline volume overload risk. Pharmacologically, fludrocortisone is approximately 70% protein-bound and has a large volume of distribution, indicating extensive tissue and intracellular penetration. These properties render it poorly dialyzable, making it a sustained blood pressure support agent throughout dialysis sessions [16]. Compared with alternative pharmacologic options, fludrocortisone is uniquely essential and irreplaceable in the management of adrenal insufficiency, whereas agents such as midodrine and droxidopa are more effective in neurogenic hypotension. In particular, droxidopa has demonstrated utility in the setting of sympathetic denervation, while vasopressin analogs may be considered in selected cases of refractory vasodilatory hypotension. [8] In this patient, the underlying adrenal insufficiency made fludrocortisone the most appropriate choice despite the theoretical risk of fluid overload. Given its pharmacologic properties, fludrocortisone may serve as a valuable adjunct to vasopressors, especially in patients transitioning off intravenous therapies.

While fludrocortisone appeared to contribute significantly to blood pressure stabilization, several concurrent factors may have influenced the patient's hemodynamic improvement. These include ongoing midodrine and hydrocortisone use, modification of the dialysis prescription, and her residual renal function. In addition, the tapering of corticosteroids over time may have allowed for some adrenal axis recovery. These variables may have contributed to the overall response and should be considered when interpreting the clinical outcome.

## Conclusions

This case highlights the successful use of fludrocortisone for refractory IDH in a critically ill patient with newly initiated hemodialysis and adrenal insufficiency. Despite the failure of standard non-pharmacological and pharmacological measures, fludrocortisone stabilized blood pressure, allowing for sustained dialysis without the need for vasopressors. Its poor dialyzability and sustained hemodynamic effects make it a promising adjunct in select patients. Early recognition of adrenal suppression and appropriate mineralocorticoid therapy may improve outcomes in this population. Given the mineralocorticoid effects of fludrocortisone, caution is warranted, and patients, particularly those with CKD, should undergo close monitoring of electrolytes and volume status to balance the hemodynamic benefits against the risk of fluid overload and electrolyte disturbances. Further research is warranted to define the role of fludrocortisone in broader dialysis cohorts.
